# Long-Term Care Registered Dietitians’ Initial Response to the COVID-19 Pandemic

**DOI:** 10.1093/geroni/igaa057.3482

**Published:** 2020-12-16

**Authors:** Julie Beitel, Allison Cammer

**Affiliations:** University of Saskatchewan, Saskatoon, Saskatchewan, Canada

## Abstract

At the outset of the global pandemic, long-term care (LTC) homes in Canada were captured in media reports as the centre of Canada’s COVID-19 epidemic. An estimated 80% of all COVID-19 deaths in Canada were associated with LTC outbreaks as of May 25, 2020. Infection control measures have swiftly changed the environment in many LTC homes for residents, workers, loved ones, and other supports. Registered Dietitians (RDs) are among the many care professionals working in LTC affected by these changes. The aim of this qualitative study was to examine the roles of RDs in supporting LTC residents during the initial phases of the pandemic. RDs faced remote practice, redeployment to address pandemic priorities, or cohorting to a sole practice site, yet were responsible for resident nutritional health. In-depth, web-based, semi-structured interviews with thirteen RDs working in LTC in a prairie province of Canada were used to explore the changes to work, challenges faced, impact on residents, and innovations in practice. The findings from this study capture nutrition and wellness-related implications of the COVID-19 pandemic within LTC homes. Examining the initial response of LTC RDs to the COVID-19 pandemic can help in planning for opportunities to support or enhance delivery of nutrition care in LTC homes, both in the context of the ongoing pandemic as well as future practice.

